# Abstracts and Gleanings

**Published:** 1886-01-20

**Authors:** 


### ABSTRACTS AND GLEANINGS.

**Prevention of Septicæmia.**—A writer in Medical and Surgical Reporter says: Five or six years ago I adopted the practice of giving chlorate of potassium to all of my puerperal patients, commencing the second day after delivery, and continuing at least five days, or, what I think a better guide, as long as there is any color to the lochial discharge.

In that time I have not had a single case of septicæmia amongst the patients I attended myself.

It is true my patients have all lived in a village of not over fifteen hundred inhabitants or in the country. It is also true that during that time there has been no epidemic of erysipelas, scarlatina, or diptheria in this vicinity.

The use of the remedy as a preventive of blood poisoning in puerpera is not original with myself, but I have extended it to my surgical cases with like good results. There may not be any power in the potassium chlorate to prevent septicæmia, but prior to the time I commenced using it such cases were not infrequent among my patients, a number was dangerously ill, and I lost a few that I treated or assisted in treating. I usually administer the remedy as follows:

℞. Chlorate potassium.................................ʒ j

Aqua pura.......................................℥ ij

M. Sig.—Half a teaspoonful every three or four hours.

It is not necessary to give it in large doses, as they are dangerous.

I have never resorted to intra-uterine injections, as some recommend, but have relied upon vaginal injections of warm water, with or without any disinfectant, as needed, as a matter of cleanliness.

The periodical fevers sometimes preceding, are ruptured as soon as possible. They are liable to arrest the lochial discharge, and that will cause septicæmia, unless prevented by proper treatment. I hope physicians, in large cities or localities where puerperal fever or septicæmia is prevalent, will try the chlorate of potassium, and report, that the profession may know whether it is a success or a failure.

**Alcoholic Stupor ("Dead Drunk.")**—(Druggists Circular) May usually be recognized by odor of breath; breathing loud and difficult. Unconsciousness prolonged. This may be confounded with apoplexy or concussion of brain from a fall or blow; generally the breath will reveal the presence of alcohol.

TREATMENT.

Give an emetic—sulphate of zinc, ipecac, common salt, ground mustard, or alum in a glass of water. Keep the patient warm.

NOTE.—Great care must be taken that the diagnosis between apoplexy or concussion and alcoholic stupor is certain, for what is proper treatment in the one case is bad treatment in the other.

**Opium Poisoning.**—(Drug Circular) Trembling, giddiness, stupor, followed by irresistible desire to sleep; prolonged unconsciousness. Pupils much contracted, breathing slow and heavy, face and eyes congested, pulse becoming slower and feebler, finally convulsions and death. Often a profuse perspiration bathes the body. Sometimes the odor of opium can be detected in the breath when the diagnosis is certain.

The amount of opium which will produce poisoning varies greatly in different persons, and also with the age. Young children are very susceptible to its influence. The time required for manifestations of the poison varies, with the solubility of the drug, from thirty minutes to two hours.

TREATMENT.

Empty the stomach at once with a stomach pump or, if that is not available, with an emetic (20 grains of sulphate of zinc, or ipecac, or ground mustard, or common salt in water). Follow the emetic with copious draughts of warm water to thoroughly rinse out the stomach. Give large amounts of the strongest coffee, and keep the patient awake by compelling him to walk, best between two stout men, in the open air. Give brandy and ammonia repeatedly in solution in warm water. Belladonna is the physiological antidote, and should be given in the proper doses (extract, gr. $\frac{1}{4}$ to $\frac{1}{2}$, or a solution of atropia, one grain to the ounce, may be made, and 15 to 20 minims injected with the hypodermic syringe). Repeat if necessary.

To empty the stomach thoroughly, keep the patient awake, and administer strong coffee and belladonna are the indications in opium poisoning. When the stupor is profound, and all remedies seem to fail, the inhalation of oxygen, and also artifical respiration, should be tried.

**Lengthening of the Child-bearing Period in American Women.**—(Detroit Lancet) Dr. Chadwick, of Boston, in the discussion of a paper on "The Natural Hygiene of Child-bearing Life," read at the late meeting of the American Gynecological Society, said that he had made some investigations in regard to the early appearance of menstruation with regard to women of various nationalities in this country. He had examined over 4,000 cases, and found that American women began to menstruate earlier than women of other nationalities examined. Women of native parentage, moreover, began to menstruate earlier than American women of foreign parentage. There has, as yet, been scarcely enough observations made in reference to the menopause, but he had found it is appearing later in American women than in the foreign-born women of this country.

If this is true, the conclusion is, that the child-bearing period is greater in American women than in women of other countries.

This increase in the child-bearing period, both at the beginning and the end, is an indication of added vigor.

The diminution in the number of children to a family may be accounted for by social influences, as is common in all civilized communities.

**Dr. Ultman's Treatment of Functional Impotence.**—(Buffalo Medical and Surgical Journal) The two forms of this affection which present themselves most frequently for treatment are: 1. Psychial impotence. 2. Impotence from two early ejaculation of the seminal fluid. They occur usually in strong, healthy, young men, and are the forms which yield to proper treatment.

The treatment is almost entirely local, as the difficulty consists in the capacity of having a normal erection. The capacity of exciting erections resides in the prostate; hence, this is the point to which treatment is to be directed. This is of several forms, all calculated to relieve the hyperæsthesia of the prostatic urethra, and excite the prostate to the production of powerful erections.

The simplest form of treatment is by passing of a steel sound into the bladder daily, leaving it there from five to ten minutes, at the same time depressing the handle so as to make pressure upon the prostate. Usually, in a few days or weeks, powerful erections are excited. The use of astringents upon the prostatic urethra is a another method. The remedies used are either tannic acid or solution of nitrate of silver. Tannic acid is inserted in small slender suppositories by the *porte remede* and deposited in the prostatic portion. Urine should not be passed for half an hour after the insertion. This treatment may be used every day, and not less frequently than every second day, till normal erections occur. Nitrate of siver is used in a five per cent. solution, and three or four drops deposited upon the prostatic portion by a deep urethral syringe, once every three or four days. During treatment, the patient should abstain from all sexual excitement.

**Transfixion of the Scrotum.**—(Medical and Surgical Reporter) Dr. Alexander Ferguson reports this rather singular accident in the British Medical Journal, October 10, 1885: He was called to see J. C., a farm-steward. He had finished the building of a corn-stack, against which had been stupidly placed a pitchfork, with the wooden handle uppermost. The shaft, which was of ash, and measuring about one inch and a quarter in diameter, entered his scrotum close to the perineum, passed upwards, and emerged on the dorsal surface, close to the penis, extruding the right testicle partially, and the left completely. No help being at hand, the poor man pluckily seized the scrotum in his left hand, and with his right withdrew the fork-handle, which had transfixed him to the extent of two and a half feet. This operation, he says, was attended with much pain. He thereafter walked home, a distance of thirty or forty yards, supporting his injured scrotum and displaced testicles.

About an hour elapsed before he saw him, and he was much struck with the disparity between the injury and the constitutional effects; the only complaint was slight nausea. He washed the testicles in a weak boracic lotion, and then restored them to their normal sites. He stitched up both wounds in the scrotum with carbolized silk, and applied a compress of boracic acid lint squeezed out of hot water, which was the only dressing. Strange to say, no rise in pulse or temperature took place.

**Cutaneous Hæmorrhage by Auto-Suggestion.**—(London Medical Press, October 7, 1885.) At the Congress Scientifique, Grenoble, a Dr. Bourru, of Rochefort, cited an extraordinary case of cutaneous hæmmorrhage by auto-suggestion which came under his observation in his hospital practice. A man, æt 22, of hystero-epileptic disposition well confirmed, was being treated for hemiplegia of the right side. Suspecting that he was a fit subject for experiment, the doctor having put him to sleep by mesmerism, said to him, "This evening, at four o'clock, when asleep, you will get up and come into my room, sit in my arm-chair, fold your arms upon your chest, and *bleed* from the nose." The command was obeyed to the letter, and a few drops of blood flowed from the *left* nostril without the slightest provocation. A few days subsequently the experimentalist traced with the blunt end of an ordinary pocket-probe upon both forearms the name of the subject, giving him the following order: "This evening you are to go to sleep, and you are to bleed from the lines I have just traced." At the stated hour the patient went to sleep, and soon appeared the scarlet lines on the *left* arm, and immediately afterwards drops of blood oozed out. Three months have passed, and the letters are still visible, but are paling. On the right side there was no mark. Since that time he has been placed in other hands, for he was sent to the hospital for epileptics, and there the experiment was renewed with similar results, before a large assemblage of doctors and magistrates. The doctor, after having traced a letter on each forearm, told the subject to bleed immediately from the lines. The man said he could not do it from the right arm, because it was paralyzed, and being told to bleed from the left, he protested, saying it hurt him very much. However, he was commanded to obey, and in a few minutes the letter became defined in red, and drops of blood flowed from it.

**A New Remedy for Sea-Sickness.**—(Maryland Medical Journal) Dr. G. W. Noad writes the British Medical Journal: Having noticed in the Journal, of September 26th, some remarks on the use of cocaine in sea-sickness, by Professor Manassein, I thought I would follow his directions in the case of my son, aged 24, who was about to sail for Calcutta, and who on former voyages, had suffered excessively from sea-sickness. I gave him a solution of hydrochlorate of cocaine (1 in 1,000). He started on October 5th, and writing from Port Said reports as follows: "Sailing on Monday, I was ill on Tuesday night and Wednesday morning but well between the attacks. Once more, when the weather was very rough, and the ship rolling terribly, I felt squeamish, but two teaspoonsful of the cocaine put me all right." He adds: "I have missed only three regular meals but had mine at these times on deck. Only one other passenger has suffered less than I have; all others have been very ill. Other voyages, I have always been the worst on board; and I think the cocaine must have the credit of the improvement." Other letters have been received, but, as no mention has been made of any return of the malady, we conclude he has remained well.

**Cocaine Intoxication.**—The subject of cocaine intoxication has recently attracted some attention in various medical circles, and a few cases have been reported in which these manifestations of this drug were noted. The London Medical Times has called attention to a case recorded by Dr. P. Heyman in the Deutsche Medicinische Wochenschrift, No. 46, which posseses some points of interest.

The patient, a boy, was suffering only from a small papillomatous growth in the larynx, which had recurred again and again after its removal, once under cocaine, in many sittings. The boy required the use of a very liberal application of the solution to the pharynx and larynx before anæsthesia could be produced. The tumor was quickly removed, but not before the boy began to show signs of intoxication. On being placed on a sofa, he lay for five hours in an apathetic half-sleepy condition with eyes open. "He had no hallucinations, or could recollect none, and when able to speak did so in a hesitating, but nevertheless intelligent manner. There was considerable difficulty in walking, the gait being very uncertain. He did not complain of hunger or of any special pain, only of general feelings of discomfort. The pupils were not dilated, but reacted normally to light. Pulse, temperature, and respiration appear to have been all slightly increased, but both the heart and lung movements were otherwise normal and regular. At a rather later stage the difficulty of walking was increased, but both the heart and lung movements were otherwise normal and regular. At a rather later stage the difficulty of walking was increased, owing to an apparent inability to control the movements of the legs. The symptoms did not pass off until ten hours after their onset, the patient then for the first time obtaining sleep, from which he awoke but little worse, apparently, for his experiences of the previous day."

**Diabetis Mellitus Successfully Treated with Boracic Acid.**—F. A. Monckton reports, in the Australasian Medical Gazette, a case of diabetes mellitus cured by the use of this drug. He says, while pointing out that the value of boracic acid as a diabetic remedy has only been proved in this one case, let me earnestly beg that those who have an opportunity of watching its effect will try it. When placed on the boracic acid the patient's urine had a specific gravity of 1.025. Seven grains of the acid were given three times a day, and at the end of ten weeks the specific gravity was 1.016; no sugar. He continues the drug, however, as it produces no unpleasant effects. No stringent dietary regulations were observed in this case.—*Louisville Medical News.*

**A Bill** is being prepared by the New York Medical Society (Medical Review) asking the State Legislature to include cocaine in the list of drugs forbidden to be sold excepting on physician's prescriptions. It is said that in New York many drug stores sell a paste made up of coca leaves and lime forming a cud similar to that used by the Peruvians as a stimulant. These preparations are in great demand.

**Tedious Labor.**—In a discussion upon a paper read by Dr. Richardson in Obstetrical Society of Philadelphia (Maryland Medical Journal), Prof. Jaggard, of Chicago, upon invitation from the Chair, remarked that morphia hypodermically had been extensively tried in the first stage of labor at Vienna and Paris, and had been discarded in the former city about six years ago, and in the latter more recently. It had been found to effect the fœtus unfavorably. One-fourth of a grain administered every four hours for some time would be attended with grave elements of prognosis. The possibility of a live fœtus remaining in the uterus forty-five days after the escape of the amniotic fluid he considered more than doubtful. Cysts sometimes form between the amnion and chorion, and the bursting of one of these may give rise to the idea of the escape of the amniotic fluid. Hydrorrhœa gravidarum, a condition dependent on a diseased condition of the decidua, is a more frequent phenomenon, and will explain many of the cases of supposed rupture of the amniotic sac.

Dr. W. T. Taylor remarked that the causes of delay in the first stage of labor were numerous. For the relaxation of rigid os he would prefer hydrate of chloral, twenty grains; one-eighth of a grain of sulphate morphia every two hours has a soothing and beneficial effect, giving rest and sleep between the pains. When the edge of the cervix is thin and wiry the morphia is especially called for. He has experienced delay from dropsy of the amnion. After a delay of six or eight hours he has ruptured membranes, and after the escape of an enormous quantity of fluid, rapid and effectual contraction supervened. Another cause of delay is posterior positions of the occiput; if a change of position can be effected labor will progress more rapidly. He has observed premature escape of the liquor amnii from ten days to two weeks before labor, and yet everything went on normally. He has met with one case of malarial poisoning. In the eighth month intense pains were experienced but there was no effect on the os. He gave five grains of quinine and ten grains of potassium bromide, and in a few hours the pain was relieved.

Dr. Longaker says that according to his experience morphia should be used guardedly. In some cases it has caused still-births. In a recent case the first stage of labor had lasted twenty-four hours, and the os was but one inch in diameter; four doses of sulphate of morphine, one-fourth of a grain each, were, by mistake of the nurse, given at intervals of fifteen minutes, dilatation and descent of the child quickly followed. As Dr. Parvin has stated, the early stage of labor consists mainly of retraction of the cervix, and early rupture of the membranes as a trouble is overated. Undeveloped pelves, of generally small diameter, cause less delay in first labors than in later ones, because in the earlier labors the abdominal muscles are strong to assist the uterine contractions; in later labors, besides having less contractile powers, their laxity allows the body of the child to fall forward and the vertex presents less favorably at the superior strait.

Dr. Trautmann. In one case under his care recently he found a well dilated os uteri and a free escape of waters; the pains ceased, ergot was given without any effect, and as the forceps were strenuously objected to, he was obliged to do nothing; after an interval of four weeks labor came on naturally and a living child was delivered. The unaccountable facts in this case are the widely dilated os, the escape of the waters and a living child four weeks later.

Dr. W. S. Stewart. Sodium bromide is useful to prevent premature labor; five drams may be divided into ten doses and one given every three hours. He has observed in one patient an apparent rupture of the membranes at five months, the fluid coming away with a constant drip; later the flow was greatest at night; this condition lasted for six weeks, when it terminated in premature labor, the fœtus was living; the fluid which came away was examined and seemed to be amniotic. He also has observed retardation of labor from falling forward of the fœtus in relaxed abdomen; when such a patient is placed on her back, labor goes on rapidly.

Dr. Chas. M. Wilson remarked that hydrorrhœa gravidarum is more frequent than is supposed, and is mistaken for premature discharge of amniotic fluid. Rigidity of the os uteri is most quickly releived by inhalations of chloroform. He has found its action more satisfactory than that of chloral and sodium bromide or ether, and safer than morphia. Postural treatment of early stages of labor is of the greatest importance; he would place the patient on the floor on her knees or haunches; holding by the back of a chair or post is often useful as it assists in fixing the respiratory muscles. He has not had good results from the local use of belladonna.

Dr. Keating spoke of some experiments he had been making. The patient was first to practice Dr. Bonwill's method of inducing partial anæsthesia by rapid long breathing for a time and then to hold the breath as long as possible. This method was found to bring on rapid and efficent pains in multiparæ with pendicular abdomen.

Dr. B. F. Baer remarked that, take it all in all, morphia hypodermically is the most valuable remedy we possess for the relief of pain and rigidity of the cervix during the first stage of labor. (Of course, it must be used within proper limits.)

Dr. E. Richardson, in closing the discussion, said that his paper was not intended to be comprehensive. His use of morphia extended only to doses of one-sixth of a grain every four hours, by the mouth, and not hypodermically. In the patient, whose history he had given, intermittent fever was developed later on and he has not the slightest doubt of malarial poisoning being the cause of the untoward symptoms during and after labor; there was no fever, no rise of temperature and, therefore, septicæmia is excluded. There can be no question as to the retraction of the cervix when the head is already in the pelvis; but when the head fits tightly into the superior strait and the cervix is jammed by it, the pressure upon the upper sac is greater than upon the lower, cut off from it by the head. Chloroform is more efficient than any other agent he had used, but it was not always to be preferred.

**Gun Shot Wounds.**—Professor Park, in a paper read before Buffalo Medical Assosiation, remarks of the treatment of gunshot wounds: If one wanted to speak in aphorisms, he might say in three words "Primary Antiseptic Occlusion." But preferring to be a little more explicit than this, I would say that, in the first place, in not more than one case out of ten is there any indication to remove the ball; while in the tenth case the indication is hardly likly to be an imperative one. Therefore, let the dangers be estimated in each case from the general condition of the patient, and let no probe, exploring instrument, nor finger be introduced. Let us remember Nussbaum's sage remark that the fate of the soldier is truly in the hands of the surgeon who first attends him; and then bear in mind that the fate of the would-be suicide may be equally in the hands of the first medical man called in.

Sir Joseph Lister, following Reyher, divides the gunshot wounds into the "fingered" and the "unfingered," the former of which need drainage and the latter nothing more than to be left alone. Supposing then that we have to deal with a wound as yet unfingered, it needs simply to be antiseptically occluded by iodoform, salicylic or sublimate cotton, and the wounded part then to be put completely at rest. The probabilities are very great that nothing more will be required, nevertheless should a chill or anything else give rise to subsequent alarm, the surgeon still has at his command every resource which he had at first, and may still feel that he gave Nature every chance to repair the wound without assistance. The only case in which exception can be taken to rigid observance of this rule are those in which unmistakable signs make it evident that some large vessel has been wounded; in such cases there is demand for the earliest and most thorough operative interference. But even then, in the writer's opinion, nothing beyond the application of a tourniquet and provisional occlusion should be attempted till everything can be done. If this plan were generally and constantly carried out we should hear of fewer fatalities, either immediate or postponed.

Last winter a young man tried to ascertain whether his revolver was or was not loaded; the result of his investigation was a wound of the hand and forearm the ball entering the palm of the hand and passing somewhere up into the forearm, its track being under the annular ligament. Dr. Briggs saw him immediately after the injury, and, forbearing any exploration, kindly sent him at once to my clinic. Here his wound was simply occluded with iodoform cotton; and the patient consenting to be guided by my advice, I left it absolutely alone, merely resting the arm on a splint. In ten days he was back at his work as a hostler, with very little stiffness remaining. Now, in this case, if I had undertaken to remove the ball, I should have had to make a comparatively extensive dissection, to make a wound which would not have healed so quickly, to have left a hand not as useful, and have introduced elements of danger which did not previously exist, aside from all æsthetic considerations of a larger scar. That would have been correct practice on the battle-field, and I fail to see why it was not equally correct in private practice.

Supposing, then, that this primary occlusion has been made, what else is necessary? Nothing, save rest for the injured part, unless symptoms arise which call for interference. Of course, I am not stultifying myself by implying that if it be very evident that clothing or palpable dirt had been carried into the wound they are not to be removed. The removal of shreds of dry goods is, of course, to be effected before the occlusion, provided anything of the kind be visible. But this is to be done at once, and nothing more, and the case then carefully watched; if subsequent fever or suppuration make it appear that repair is not going on as it should, then the occlusive pad may be taken off and the wound washed out and drained, or it may be enlarged and explored as circumstances may seem to require.—*Physicians Magazine.*

**Oil of Eucalyptus and Oil of Turpentine in Membranous Croup.**—Dr. F. A. Johnson, in California Medical Journal, writes: Allow me to briefly report a severe case of membranous croup cured by me without tracheotomy. To perform this very hazardous operation would have been certain death to my little patient, on account of vascularity of the thyroid gland; I preferred to let the patient die a natural death. Feeling that all was not done that could be, and having but a few moments to work in, I took an atomizer, charged with oil of eucalyptus and oil turpentine aa. q. s. and with the mouth well opened, I sprayed the mouth and throat with this mixture every 15 minutes for at least two hours, and from the first the patient breathed easier. At the same time I gave my patient carbonate of ammonia in brandy, aa., one teaspoonful as often as every 15 minutes, alternated with the spray. At the end of two and one-half hours the little sufferer was running about the room. I had discovered, several weeks previous, that the two oils would dissolve India-rubber, and made a record of the fact that if I ever had a case of this terrific disease, or of diphtheria, I would give the two drugs a trial. I will here state that as powerful and volatile as the two drugs are they have no bad effect upon the mucous membrane, but will dissolve the membrane very rapidly. Try it my brother physicians, and report your success in your respective medical journals.

I do believe, beyond a doubt, that by a judicious use of these two oils, diphtheria and membranous croup can be cured without the use of the knife.

**Migraine—What is its Cause?**—This curious trouble, while it does not destroy life, often seriously cripples it in its usefulness. Various views are held as to its cause. Dr. Alfred Drysdale (The Practitioner) claims that it is due to the accumulation in the blood of a peculiar poisonous substance, possibly allied to creatin and creatinin. We know that poisons have an elective affinity for certain organs and parts of the body, and we must suppose that this substance selects the great nerves at the base of the brain—the optic, the ophthalmic, and the pneumogastric. The reason for supposing that the substance is allied to creatin is the close resemblance of the symptoms produced by it to those of uræmic poisoning, which is known to be dependent upon the presence of these compounds in the blood. The blindness, headache, vomiting, and subsequent stupor bordering upon coma in migraine are indistinguishable, except in degree, from the same symptoms in uræmia. The poison, whatever its nature be, must speedily produce its own elimination, otherwise there would be no reason for the subsidence of the attack.

To prevent the accumulations of this poison, he advises exercise, regular and pushed to the point of fatigue. To mitigate the attack after it has commenced, he has gained the best results from nitroglycerine.—*Detroit Lancet.*

**Treatment of the Opium Habit.**—(Druggists Circular) Dr. J. B. Mattison, in a recent monograph on the Treatment of Opium Addiction, characterizes the old Levinstein method of complete and immediate withdrawal of the drug as a "barbarous plan," "utterly inexcusable," and "positive malpractice." He recommends, says the Medical Record, September 26th, a mean between abrupt cessation and the very gradual decrease which tends to exhaust the patience of the patient. He relies on bromides, preferably sodium bromide, beginning with a "preliminary sedative." The initial dose is 60 grains twice daily, at intervals of twelve hours, increasing the amount 20 grains each day, and continuing it five to seven days, until reaching a maximum dose of 100 to 120 grains twice in twenty-four hours. In the meantime the opiate is decreased, at first from one-fourth to one-third the usual amount taken daily, and a. little less rapidly at the last, so that from the eighth to the tenth day it is entirely abandoned. In the case of weak patients the above treatment is preceded by a tonic course. Bromidism, he says, if produced, soon subsides, and leaves no unpleasant after-effects.

**The Treatment of Frost-bitten Fingers and Toes.**—Dr. Lapatin, in the Proceedings of the Caucasian Medical Society, advises that fingers and toes which have been slightly frost-bitten, and which subsequently suffer from burning, itching, and pricking sensations, should be painted, at first once and afteward twice a day, with a mixture of dilute nitric acid and peppermint water in equal proportions. After this application has been made for three or four days the skin becomes darkened and the epidermis is shed, healthy skin appearing under it. The cure is effected in from ten to fourteen days. The author has found this plan very effectual among soldiers who were unable to wear their boots in consequence of having had frozen feet. They were in this way soon rendered capable of returning to military duty.—*British Medical Journal.*

**For Spinal Tenderness.**—(Medical World) Torino (of Italy) noticed, in many cases of vomiting of pregnancy, that there were tender spots over the dorsal vertebræ, indicating spinal anæmia or irritation. He prescribed phosphate of zinc (about grain 1-12) and extract nux vomica (grain ½) three times a day, with almost immediate benefit.

**The Action of Antipyrine upon the Croupous Pneumonia of Children.**—(Deutsche Medical Zeitung) Accurate observations upon the value of this drug were recently made upon five children ranging between the ages of four and eight years, who were suffering from croupous pneumonia. It was administered in the form of powder dissolved in water, and was received by the children without repugnance, also being well tolerated. In twenty-five doses in which it was given, vomiting was excited only twice; in a few other cases there was slight nausea. About three hours after its administration the temperature had in most cases declined two degrees. In some cases it went below the normal, but never with any symptoms of collapse. The pulse usually become stronger, but its abnormal frequency did not diminish at the same rate with the temperature. As compared with kairine it was observed that antipyrine produced a more gradual declension of temperature. The scale of dosage which was adopted was the following: To children from six months to a year old, every three hours until three doses had been given, were administered two-tenths of a gram. From one to three years, every two or three hours, three-tenths of a gram. From four to five years, every two hours, three-tenths to four-tenths of a gram. From six to eight years, every two hours, five-tenths to six-tenths of a gram, From ten to twelve years, every hour, from six-tenths to seventy-five hundredths of a gram. In no case should more than three doses per day be given. The same drug was also given to four healthy children, the result being that the average decline of the normal temperature was from one to one and a half degrees, and the greatest variations from the normal always took place during the hours of the night.—*Archieves Pediatrics.*

**Treatment of Fainting.**—(Druggists Circular) The patient should be laid in a horizontal position, with the head a little lower than the shoulders, that gravitation might aid the blood in reaching the brain ; the erect position is dangerous for a fainting person. All clothing which constricts the neck or the chest should be removed or thoroughly loosened. There should be an abundance of fresh air about the patient ; if in a close room, he should be carried into the open air. Cold water should be forcibly sprinkled upon the face and upon the bared chest, and friction applied to the hands and arms. Stimulating vapors, as ammonia and eau de cologne, may be applied to the nostrils. An excellent application is a plate dipped in hot water, and placed over the pit of the stomach, or over the heart. All else failing, electricity may be carefully tried, one pole being applied to the back, and the other in the region of the heart. Fainting which results from excessive hemorrhage must be treated with perfect quiet and stimulants, carefully administered.

**Antipyrine.**—(New York Medical Journal) M. Dumolard gives preference to the following formula: Antipyrine, 20 parts; Jamaica rum, 30 parts; syrup and water, each 150 parts. In typhoid fever he gives a teaspoonful three times a days.

**Asthma as Related to Skin Diseases.**—Dr. L. Duncan Bulkley, in a paper on this subject, read before the British Medical Association (British Medical Journal), from the results of an extended study, concludes as follows:

1. Asthma has been observed in patients with certain diseases of the skin in such a manner as to indicate some occasional relationship between the two.

2. Asthma does not occur, probably, in more than one per cent. of patients with diseases of the skin, and those mainly of the class known as exudative or inflammatory disorders.

3. This occurrence of asthma in skin-patients cannot be looked upon as a coincidence, nor is the skin disease to be regarded as a cause of the asthma; but both the skin and bronchial difficulty depend upon the same internal cause, which may be nervous in origin, or may result from some altered condition of the blood.

4. While the theory of the dependence of asthma on a state of spasm of the muscular element of the bronchial tubes has very strong evidence in its favor, it is still possible that the paroxysm of asthma may be occasioned by sudden and evanescent swelling of the mucous membrane of the bronchioles, partaking more or less of the wheals of urticaria, occurring both on the mucous membrane of the mouth and on the skin.—*Louisville Medical News.*

**Alcohol as a Hypnotic in Fevers.**—(Medical World) Alcohol is principally useful as an inducer of sleep when insomnia is associated with extreme bodily exhaustion, such as occurs in advanced stages of the infective fevers. Here the heart is weakened, the pulse is feeble and accelerated, the muscular apparatus is wasted and irritable, and the blood is diminished in volume and tends to accumulate in the venous channels. Under such conditions, there is usually delirium. An ounce of good brandy, with milk and egg—"egg nog",—repeated *pro re nata*, will often remedy this and assure refreshing sleep; the temperature will fall, the tongue will moisten; and the delirium will diminish or depart altogether. These results, however, are not always observed, and bromides have to be substituted.

**Glyclosuria.**—(Medical and Surgical Reporter) Dr. A. M. Duncan, of Hamler, Ohio, writes to the Medical Record that Dr. Alvord, a retired practitioner of that place, who is a sufferer from glycosuria, finds more relief from a diet of pure buckwheat-flour cakes than from anything else. While he adheres to this food the urine becomes nearly normal in quantity and quality, there is no gastric distress, and the pain in the eyes—nearly destroyed by chronic iritis—is markedly relieved. On resuming the use of wheat bread and other starchy foods, the symptoms become aggravated, to be again relieved upon a return to buckwheat.

**"Populin,"** (Medical World) an alkaloid obtained from the bark of white poplar, or "quaking aspen," is said to act like a charm in painful micturition and scalding. The dose ranges from two to four grains.

**External Use of Chloral Hydrate.**—(Paris Medical) Nicolai has obtained excellent results in the treatment of night sweats, in phthisis and other diseases, by the use of chloıal hydrate externally, as follows:

R. Chloral Hydrate..........................dr. 2

Brandy, } aa..................................oz. 4.

Water, }

M. Sig. Lotion.

The patient is sponged off with this lotion every night, and if this does not effect a cure, a shirt should be soaked in the solution and dried, which the patient is to wear at night. This remedy is especially valuable in treating the night sweats of children. Sometimes, three or four applications of this lotion will entirely relieve cases that have been troubled with night sweats for several weeks.

**Treatment of Acute Rheumatism.**—(Maryland Medical Journal.) Dr. R. H. Fox states in the British Medical Journal, October 10, 1885, that in a severe case of rheumatism in which salicylate of sodium, potassium, quinine, colchicum, and liniments had all failed to relieve the fever and pain, the relief was immediate after sponging with cold water and quickly drying the skin afterward. Although this is no new treatment, it is one which requires some courage to practice, and yet may be well adapted to certain severe cases in which the salicylic remedies are ineffectual.

**M. Pierre Vigier** (Gazette Hebdomadaire, February 6, 1885,) states that for injection in gonorrhœa the lactate of quinine is the best preparation of quinine, owing to its greater solubility. His formula is lactate of quinine 1 gramme, distilled water 75, and glycerine 25 grammes. About five grammes should be injected three or four times a day. M. Vigier takes the opportunity of recommending practitioners to more and more employ the lactate in preference to any other preparation, whether for internal, or hypodermic use. It is the salt which is best adapted for every therapeutical application.—*Medical and Surgical Reporter.*

**Dr. Hand,** in the Northwestern Lancet, reports an epidemic of goitre among the inmates of the Minnesota State Reform School. Forty-four out of one hundred and forty were suffering at the time of the doctor's observation. It was thought that the origin of the trouble lay in the flour from which the bread was made. The usual treatment with a change in the flour was speedily followed by a cure of most of the cases.—*Detroit Lancet.*

**A Little Girl** in Watertown, N. Y., who was dying of scarlet fever, desired to send a kiss to a little playmate in another town. She kissed the letter, which was sent by mail to the little friend, who, wholly unaware of the danger incurred, kissed the letter as a message from her dead friend. In a few days she herself died from scarlet fever contracted by means of this kiss.—*Courier of Medicine.*

**We**, says the Medical Abstract, are sorry to see that Professor Meandra, of Outopos, has left the direct walks of medical science and become skeptical as to the efficiency of the microbe in all cases. In his great work on the "Destiny of Man," he says: "We see tottering to its fall the old-fogy notion that man is a responsible being, the mighty endeavors of ten thousand doctors, armed with microscopes and bottles of vesuvin, have demonstrated the energy of the ubiquitous bacillus, and have proved—beyond the shadow of a doubt—that all sickness, disease and crime, owes its origin to minute germs which float ceaselessly in the atmosphere seeking for a nidus." Looking a few feet further down in the voluminous MS. of this great thinker, we find the following: "We see the graduated metamorphosis of our prisons into hospitals, and find heredity and the bacillus making way with the last vestige of freedom of the will; the crimnal, like the drunkard, is no longer amenable to the law, he has simply an unfortunate diathesis. But a change may come, the mutations of humanity are constant, and the court room of the future may show us a scene like the following:

Judge. "What is the matter with the prisoner?"

Officer. "Found him sitting on a stoop holding on to his head."

"Where were you last night, prisoner?"

"I was at home, sir; I don't drink, I had been walking in the sun and felt dizzy."

"Where was your umbrella?"

"I haven't got one, sir."

"Don't you know that it is against the law to walk in the sun without an umbrella? You may go this time, but I caution you to buy an umbrella or keep on the shady side in future."

"Next!"

"Found this man clinging to a lamp-post, rubbing his abdomen and groaning."

"What's the matter, prisoner?"

"I had just been to dinner at Robinson's restaurant and felt sick."

"What did you have for dinner?"

"Beefsteak and mince pie."

"Officer, did you make a microscopical examination of the mince pie at Robinson's?"

"I did, sir; there was no fungoid growths visible under a power of 250 diameters, the fat stood Margarine's test, and the record showed that the pies had not been made for more than four days. The prisoner eat a whole pie of legal dimensions."

"A whole pie! don't you know, prisoner, that it's against the law to eat over two cuts of mince pie at one sitting? I fine you ten dollars, and will give you sixty days if I see you here again, there is getting to be too much sickness in this district. Clerk, if that cholera case is able to stand up send him in here for trial."

**Antipyrine.**—M. Dumolard gives preference to the following formula: Antipyrine, 20 parts; Jamaica rum, 30 parts; syrup and water, each, 150 parts. In typhoid fever he gives a teaspoonful three times a day.—*New York Medical Journal.*

